# MiR-202-5p Inhibits RIG-I-Dependent Innate Immune Responses to RGNNV Infection by Targeting TRIM25 to Mediate RIG-I Ubiquitination

**DOI:** 10.3390/v12030261

**Published:** 2020-02-27

**Authors:** Wei Liu, Yilin Jin, Wanwan Zhang, Yangxi Xiang, Peng Jia, Meisheng Yi, Kuntong Jia

**Affiliations:** 1 School of Marine Sciences, Sun Yat-sen University, Guangzhou 510275, China; 2 Southern Marine Science and Engineering Guangdong Laboratory (Zhuhai), Zhuhai 519082, China; 3 Guangdong Provincial Key Laboratory of Marine Resources and Coastal Engineering, Guangdong 510275, China

**Keywords:** MiR-202-5p, TRIM25, RIG-I, ubiquitination, red spotted grouper nervous necrosis virus

## Abstract

The RIG-I-like receptors (RLRs) signaling pathway is essential for inducing type I interferon (IFN) responses to viral infections. Meanwhile, it is also tightly regulated to prevent uncontrolled immune responses. Numerous studies have shown that microRNAs (miRNAs) are essential for the regulation of immune processes, however, the detailed molecular mechanism of miRNA regulating the RLRs signaling pathway remains to be elucidated. Here, our results showed that miR-202-5p was induced by red spotted grouper nervous necrosis virus (RGNNV) infection in zebrafish. Overexpression of miR-202-5p led to reduced expression of IFN 1 and its downstream antiviral genes, thus facilitating viral replication in vitro. In comparison, significantly enhanced levels of IFN 1 and antiviral genes and significantly low viral burden were observed in the miR-202-5p^-/-^ zebrafish compared to wild type zebrafish. Subsequently, zebrafish tripartite motif-containing protein 25 (zbTRIM25) was identified as a target of miR-202-5p in both zebrafish and humans. Ectopic expression of miR-202-5p suppressed zbTRIM25-mediated RLRs signaling pathway. Furthermore, we showed that miR-202-5p inhibited zbTRIM25-mediated zbRIG-I ubiquitination and activation of IFN production. In conclusion, we demonstrate that RGNNV-inducible miR-202-5p acts as a negative regulator of zbRIG-I-triggered antiviral innate response by targeting zbTRIM25. Our study reveals a novel mechanism for the evasion of the innate immune response controlled by RGNNV.

## 1. Introduction

The innate immune system is the first defense line that recognizes pathogen-associated molecular patterns (PAMPs) by pattern recognition receptors (PRRs) against microbial pathogen invasion [1]. Retinoic acid inducible gene-I (RIG-I)-like receptors (RLRs), comprised of RIG-I, melanoma differentiation–associated gene (MDA) 5, and laboratory of genetics and physiology (LGP) 2, recognize non-self-signatures of viral RNAs in the cytosol of cells and activate their downstream signal transduction to trigger host interferon (IFN) responses and eliminate invading viruses by induction of a wide range of IFN-stimulated genes (ISGs) [2]. However, hosts and viruses have developed a variety of mechanisms to modulate the RLRs signaling pathway to avoid excessive IFN production and antagonize such innate antiviral responses via targeting multiple steps in the RLRs signaling pathway, respectively. For instance, west Nile virus non-structural protein 1 protein antagonized IFN β production by targeting RIG-I and MDA5 [3]. Ring finger protein (RNF) 122 targeted RIG-I for proteasomal degradation to inhibit RLRs-mediated IFN production [4].

Nervous necrosis virus (NNV) is one of the major pathogens that infects fresh and marine fish and causes serious economic losses worldwide [5]. Increasing evidences showed that the RLRs signaling pathway was involved in NNV infection. For example, zebrafish RIG-I plays an essential role in group II type I IFN induction during NNV infection [6]. Our previous studies also suggested that the RLRs signaling pathway is activated during red spotted grouper nervous necrosis virus (RGNNV) infection in sea perch (*Lateolabrax japonicus*) and its key components possessed anti-RGNNV activities [7,8]. It has been known that viruses can escape from host recognition by degradation of RLRs or interference with the RLRs signaling to establish persistent infections [9]. Several studies have addressed NNV persistent infections in many fish including zebrafish, grouper, and barramundi [10,11] despite the IFN response being induced in the persistent infection individuals, indicating NNV could evade or counteract the host IFN system. However, the molecular mechanisms through which NNV evade or inactivate the complex signaling pathway of host innate immunity are not completely understood.

It has been known that some viruses utilize their encoded proteins to evade the restriction of RLRs signaling pathway and even hijack host factors to facilitate their infections in mammals [2]. MicroRNAs (miRNAs), as post-transcriptional regulators, play critical roles in various biological processes, such as cell proliferation and differentiation, cell cycle and apoptosis, and immunoregulation [12,13,14]. Increasing evidence has revealed that miRNAs are implicated in numerous viral infections as negative or positive factors of antiviral innate immunity pathways, including RLRs signaling pathway. MiRNA-146a inhibits RIG-I-dependent type I IFN production by targeting TNFR-associated factor (TRAF) 6, IL-1Rassociated kinase (IRAK) 1, and IRAK2 [15]. MiRNA-3570 negatively regulates the RIG-I-dependent innate immune response to rhabdovirus in miiuy croaker (*Miichthys miiuy*) by targeting mitochondrial antiviral signaling protein (MAVS) [16]. However, how RGNNV utilizes miRNAs for immune evasion remains unknown. 

In this study, we identified miR-202-5p as a new negative regulator of RLRs signaling pathway and defined a novel function for miR-202-5p to facilitate RGNNV infection. Our findings reveal a novel mechanism of RGNNV to inhibit RLRs signaling pathway by miR-202-5p and will help to develop new treatments for viral nervous necrosis disease.

## 2. Materials and Methods 

### 2.1. Ethics Statement

All procedures with zebrafish were approved by the Ethics Committee of Sun Yat-Sen University (protocol no. 20200110008, approval date: 11 October 2019) and the methods were carried out following the approved guidelines.

### 2.2. Fish Strains, Cell Lines, and Reagents

Zebrafish wild type AB line was purchased from the China zebrafish resource center. MiR-202-5p^-/-^ zebrafish were generated in our laboratory previously [17]. Fish were raised at 28 °C with 10 h darkness and 14 h light and were fed with commercial pellets twice a day. All embryos were collected after natural spawning and staged as previously reported [18]. 

ZBE3 cells derived from zebrafish embryos were cultured at 28 °C as previously described [19]. HEK 293T cells were maintained in Dulbecco’s modified Eagle’s medium (DMEM) supplemented with heat inactivated 10% FBS (Invitrogen, California, USA), pyruvate glucose, and L-glutamine (Gibco, California, USA), at 37 °C under a humidified atmosphere of air containing 5% CO_2_. 

The anti-Flag (M20008) and anti-HA antibodies (M20013) were purchased from Abmart (Guangzhou, China). Anti-α-Tubulin (ab15246) and anti-GFP antibodies (G1544) were purchased from Abcam (London, Britain) and Sigma (Missouri, USA), respectively. The secondary antibodies goat anti-rabbit IgG-HRP and goat anti-mouse IgG-HRP were purchased from Invitrogen.

miRNA mimics for miR-202-5p, hsa-miR-202-5p, and negative controls of miRNA mimics (Ctrl-m) were purchased from Ribobio (Guangzhou, China).

### 2.3. Viral Challenge

For in vitro infection, ZBE3 cells were infected with RGNNV at a multiplicity of infection (MOI) of 1 for 12 h and 24 h, respectively. Then, the infected cells were collected for RNA isolation. 

To detect the expression of miR-202-5p in vivo, 1 nL of RGNNV (10^8^ TCID_50_/mL) was injected into 1-cell stage embryos of wild type (WT) zebrafish with a microinjection machine (ZGBPCO1500). An equal number (40 embryos) of control or infected embryos were collected for RNA isolation at 24 and 48 h post fertilization (hpf).

Fish were divided into experimental and control groups (30 per group). Adult wild type and miR-202-5p^-/-^ zebrafish were infected intraperitoneally with 50 μL of RGNNV (10^6^ TCID_50_/mL) or PBS, respectively. Randomly, three fish from challenged wild type and miR-202-5p^-/-^ groups were collected at 24 and 48 h post injection, respectively. Subsequently, RNA from these fish was extracted to detect the expression of antiviral genes and *RNA-dependent RNA polymerase* (*RDRP*) by quantitative real-time PCR (qRT-PCR). 

### 2.4. Prediction of MiR-202-5p Target Genes

Target genes of miR-202-5p (5′-UUCCUAUGCAUAUACCUCUUUG-3′) and Hsa-miR-202-5p (5′-UUCCUAUGCAUAUACUUCUUUG-3′) were analyzed for sites complementary to the miR-202-5p seed sequence (UUCCUAUG) by using both PITA (http://genie.weizmann.ac.il/pubs/mir07/mir07_data.html) and miRanda (http://www.microrna.org/microrna/home). Genes predicted by both PITA and miRanda were considered as potential target genes of miR-202-5p.

### 2.5. Plasmid Construction

The cDNA fragment of zebrafish tripartite motif-containing protein 25 (zbTRIM25) (GenBank accession no. NM200175.1) including the sequences of ORF and partial 3′UTR, was cloned into pCMV-Flag vector (Invitrogen). The ORF of zbTRIM25 was sub-cloned into pCMV-Flag vector (Invitrogen) to generate recombinant plasmid pCMV-Flag-zbTRIM25. Full-length zbRIG-I and zbRIG-I deletion mutant zbRIG-I-2CARD and zbRIG-I-RD were constructed in our laboratory [20]. HA-K63Ub plasmid was purchased from Rebio (Shanghai, China). To construct 3′-UTR luciferase reporter plasmid of zbTRIM25, zbTRIM25 3′-UTR fragment (300 bp) containing the putative miR-202-5p binding site was amplified and cloned into psiCHECK2 vector (Promega, Wisconsin, USA). To construct the mutant 3′-UTR luciferase reporter plasmid of zbTRIM25, we used an over-lap PCR approach to introduce five base pair mutants in the seed region of miR-202-5p binding site. 

The wild or mutant 3′-UTR luciferase reporter plasmids of human tripartite motif-containing protein 25 (TRIM25) were constructed as described above. The primer sequences were listed in Table S1. All plasmids were sequenced to verify integrity.

The zebrafish IFN1 promoter plasmid (*pGL3-DrIFN 1-pro-Luc*) was kindly provided by Prof. Yibing Zhang at the Institute of Hydrobiology, Chinese Academy of Sciences.

### 2.6. RNA Isolation and qRT-PCR 

ZBE3 cells in 6-well plates were transfected with miR-202-5p mimics or Ctrl-m (700 ng) by Lipofectamine 3000 (Invitrogen) for 24 h, then infected with RGNNV at a MOI of 1 for 24 h. The cells were collected for RNA isolation.

Total RNA was isolated using Trizol reagent (Invitrogen) according to the manufacturer’s instructions. The first-strand cDNA was synthesized using PrimeScript™ 1st Strand cDNA Synthesis Kit (Takara, Dalian, China) and was diluted 6 folds before qRT-PCR analysis. The efficiencies for all primers were greater than 95%. QRT-PCR analyses of *zbTRIM25*, *zbRIG-I*, *RDRP*, RLRs signaling pathway related genes (*MAVS*, *TRAF3*, *IRF3*, and *IFN 1*) and ISGs ([*Myxovirus resistant a*] *MXa*, [*dsRNA-dependent protein kinase*] *PKR*, and *ISG15*) were performed as previously described [21]. The relative gene expression was normalized with *18s rRNA* using 2^-ΔΔCt^ methods. Expression analysis of miR-202-5p was performed using Mir-XTM miRNA First Strand Synthesis Kit (Takara) as previously described [22]. Data were shown as mean ± SD from three independent experiments in triplicate. The primer sequences were listed in Table S1.

### 2.7. Dual Luciferase Reporter Assay

For the miR-202-5p target efficiency assay, HEK 293T cells in 24-well plates were transiently co-transfected with plasmids (wild-type or mutant psiCHECK2-3′UTR-zbTRIM25) (10 ng) and miR-202-5p mimics or Ctrl-m (175 ng) using Lipofectamine 3000 according to the manufacturer’s instructions. Cells were lysed at 48 h post transfection, and the luciferase activities were measured by a dual-luciferase reporter assay system (Promega). The relative luciferase activities were determined by normalizing firefly activity to renilla activity. Data were shown as mean ± SD from three independent experiments in triplicate. 

To detect the effect of hsa-miR-202-5p (a homologous miRNA to miR-202-5p) on human TRIM25 promoter, HEK 293T cells were co-transfected with hsa-miR-202-5p mimic or Ctrl-m (175 ng) in combination with wild-type or mutant *psiCHECK2-3′UTR-hsTRIM25* (10 ng) for 48 h. The relative luciferase activities were measured as described above.

To test zebrafish IFN 1 promoter activity, HEK 293T cells seeded in 24-well plate were transfected with *pCMV-Flag* or *pCMV-Flag-zbTRIM25* plasmids (100 ng) together with miR-202-5p mimics or Ctrl-m (175 ng) as well as *pGL3-DrIFN 1-pro-Luc* (100 ng) and *pRL-TK* (25 ng) vectors for 24 h. After treatment with poly I:C (5 mg/mL) for 24 h, the relative luciferase activities were measured as described above. 

HEK 293T cells in 24-well plates were transfected with 250 ng of *pGL3-DrIFN1-pro-Luc* plasmid or pGL3-Basic empty vector with 25 ng of *pRL-TK* vector (Promega). In addition, the following plasmids were co-transfected: 125 ng of *pCMV-Flag-zbTRIM25*, 125 ng of mutant zbRIG-I or empty control plasmids, 175 ng of miR-202-5p mimics or Ctrl-m. Then, the cells were treated with poly I:C (5 mg/mL) and lysed for luciferase assay as described above. At least three independent experiments were performed.

### 2.8. Co-Immunoprecipitations (Co-IP) and Ubiquitination Assays 

Co-IP and ubiquitination assays were performed as described previously [20,23]. HEK 293T cells in 10-cm plates were co-transfected with 10 μg of different plasmid combinations (4 μg of Flag-zbTRIM25, 4 μg of pEGFP-RIG-I, and 2 μg of HA-K63Ub) and 3.5 μg of miR-202-5p mimics or Ctrl-m. Then, the cells were lysed on ice with lysis buffer for 15 min at 48 h after transfection. The cell lysates were then immunoprecipitated with anti-GFP antibodies. Immunoprecipitates or whole-cell lysates were immunoblotted with anti-HA, anti-GFP, anti-Flag, and anti-Tubulin antibodies, respectively. 

### 2.9. Statistics Analysis

All statistics were calculated using SPSS version 20. Differences between control and treatment groups were assessed by one-way ANOVA. *p* < 0.05 was considered a statistically significantly difference.

## 3. Results

### 3.1. MiR-202-5p Expression is Up-Regulated Post RGNNV Infection In Vivo and In Vitro

**Figure 1:**
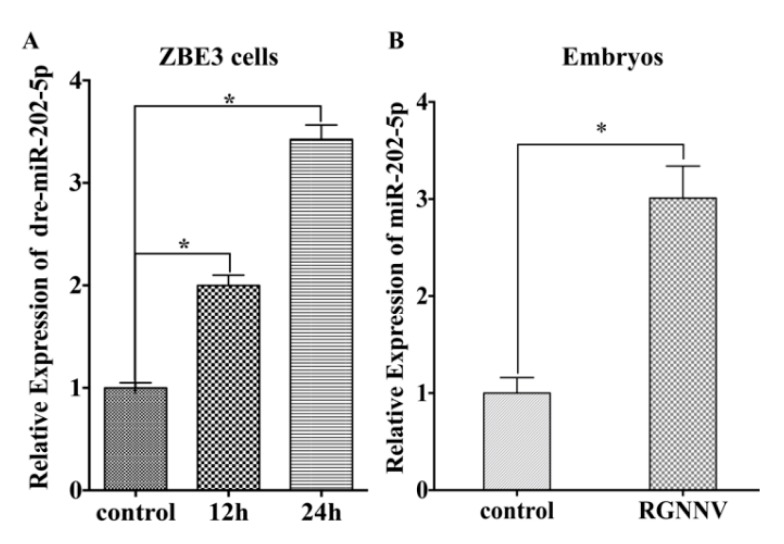
miR-202-5p is upregulated in zebrafish during red spotted grouper nervous necrosis virus (RGNNV) infection. (**A**) ZBE3 cells were infected with RGNNV for different periods. (**B**) Zebrafish embryos were infected with RGNNV for 24 h. The expression of miR-202-5p was tested by qRT-PCR. The relative expression of miR-202-5p was normalized with *18s rRNA* and represented as fold induction relative to the transcription level in the control, which was regarded as 1. Asterisks indicate the significant differences between groups (*: *p* < 0.05).

Our previous study demonstrated that ZBE3 cells were susceptible to RGNNV infection [19]. To investigate the roles of host miRNAs during RGNNV infection, a miRNA profile was obtained from the mock and RGNNV infected ZBE3 cells to analyze the relationship between miRNAs and RGNNV infection. MiR-202-5p, which is on the list of the top 10 most up-regulated miRNAs post RGNNV infection (its fold change was 5.32), was selected for further study. To further confirm the up-regulation of miR-202-5p during RGNNV infection, qRT-PCR analysis was performed in RGNNV-infected ZBE3 cells. Results showed that miR-202-5p was significantly increased upon RGNNV infection in a time dependent manner in vitro (Figure 1A). Meanwhile, we also investigated the expression of miR-202-5p in RGNNV infected zebrafish embryos at 24 h, and the results were concordant with ZBE3 cells (Figure 1B).

### 3.2. MiR-202-5p Suppresses the Expression of Antiviral Genes and Promotes RGNNV Replication

**Figure 2:**
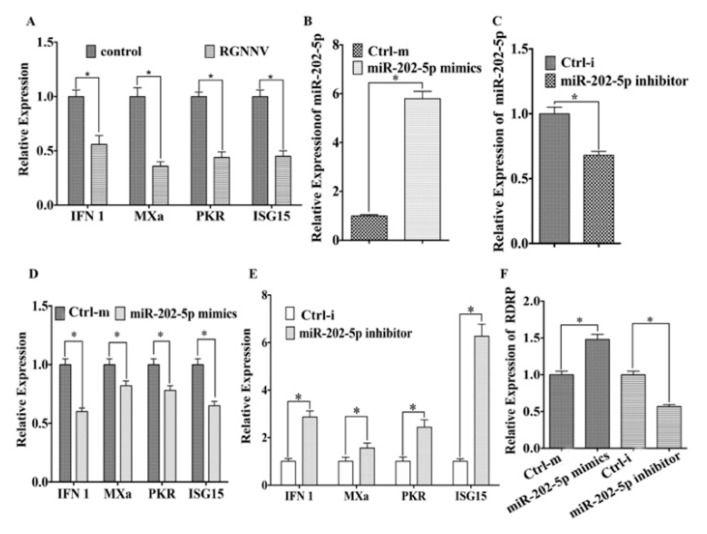
miR-202-5p inhibits antiviral genes expression and promotes RGNNV infection in vitro. (**A**) The expression of *IFN 1*, *PKR*, *MXa*, and *ISG15* mRNA post RGNNV infection. ZBE3 cells were infected with RGNNV (MOI=1) or uninfected (control) for 24 h, total RNA was extracted for qRT-PCR assays. (**B**,**C**) Effects of miR-202-5p mimics and inhibitor on expression of miR-202-5p. ZBE3 cells were transfected with miR-202-5p mimics and control mimics (Ctrl-m) (**B**) or with miR-202-5p inhibitor and inhibitor control (Ctrl-i) (**C**) for 48 h, and then total RNAs were extracted to examine the expression of miR-202-5p. (**D–F)**, Effects of miR-202-5p mimics and inhibitor on expression of *IFN 1*, *PKR*, *MXa*, *ISG15,* and RGNNV *viral RNA-dependent RNA polymerase* (*RDRP*). ZBE3 cells were transfected with miR-202-5p mimics and Ctrl-m (**D**) or with miR-202-5p inhibitor and Ctrl-i (**E**) for 24 h before RGNNV infection. At 24 h post RGNNV infection, the expression levels of *IFN 1*, *PKR*, *MXa*, *ISG15* (**D**,**E**) and *RDRP* (**F**) were detected by qRT-PCR. The relative expression levels were normalized with *18s rRNA* and represented as fold induction relative to the transcription level in the control, which was regarded as 1. Asterisks indicate the significant differences between groups (*: *p* < 0.05).

To clarify the role of miR-202-5p during RGNNV infection, we examined the effects of miR-202-5p on the immune response to RGNNV in ZBE3 cells. As shown in Figure 2A, RGNNV inhibited the expression of *IFN 1*, *PKR*, *MXa*, and *ISG15* in ZBE3 cells. The transfection of miR-202-5p mimics increased miR-202-5p expression about 4.6-fold (B), whereas miR-202-5p inhibitor significantly decreased its expression level (C). Furthermore, the expression levels of *IFN 1*, *PKR*, *MXa*, and *ISG15* were significantly downregulated and upregulated by miR-202-5p mimics or miR-202-5p inhibitor in ZBE3 cells compared to the control group post RGNNV infection, respectively (Figure 2D,E). Meanwhile, miR-202-5p mimics promoted RGNNV replication, whereas miR-202-5p inhibitors suppressed RGNNV replication (Figure 2F). 

**Figure 3:**
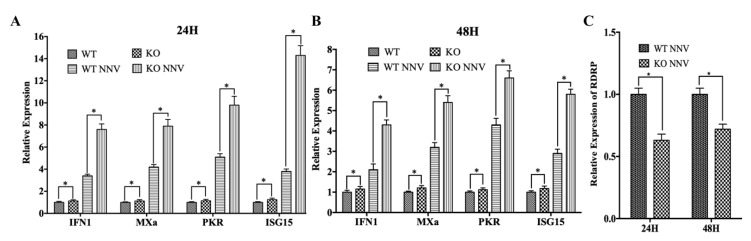
miR-202-5p knockout inhibits antiviral genes expression and promotes RGNNV infection in vivo. Adult wild type (WT) and miR-202-5p^-/-^ zebrafish (KO) were intraperitoneally injected with RGNNV or the same volume of PBS. Twenty-four and forty-eight hours later, zebrafish were harvested and expression levels of *IFN 1*, *MXa*, *PKR*, and *ISG15* (**A**,**B**) and *RDRP* (**C**) were analyzed by qRT-PCR. The relative expression levels were normalized with *18s rRNA* and represented as fold induction relative to the transcription level in the control (WT), which was regarded as 1. Asterisks indicate the significant differences between groups (*: *p* < 0.05).

To further determine the role of miR-202-5p in vivo, we performed RGNNV challenge experiments in miR-202-5p^-/-^ and wild type zebrafish, respectively. As shown in Figure 3A,B, the mRNA levels of *IFN 1* and its downstream antiviral genes (*PKR*, *ISG15*, and *MXa*) were mildly upregulated in miR-202-5p knockout zebrafish (KO) compared to that in wild type zebrafish (WT) without RGNNV infection. Upon RGNNV infection, the expression levels of these genes increased markedly in both wild type and miR-202-5p^-/^ zebrafish, and their expression levels were significantly higher in miR-202-5p^-/-^ zebrafish than that in wild type zebrafish at 24 and 48 hpi, respectively. Correspondingly, the viral replication levels were significantly lower in miR-202-5p^-/-^ zebrafish than that in wild type zebrafish (Figure 3C), suggesting miR-202-5p deletion suppressed RGNNV replication. Taken together, all the evidence strongly demonstrates that miR-202-5p suppresses the expression of antiviral genes and promotes RGNNV replication.

### 3.3. MiR-202-5p Targets zbTRIM25

**Figure 4:**
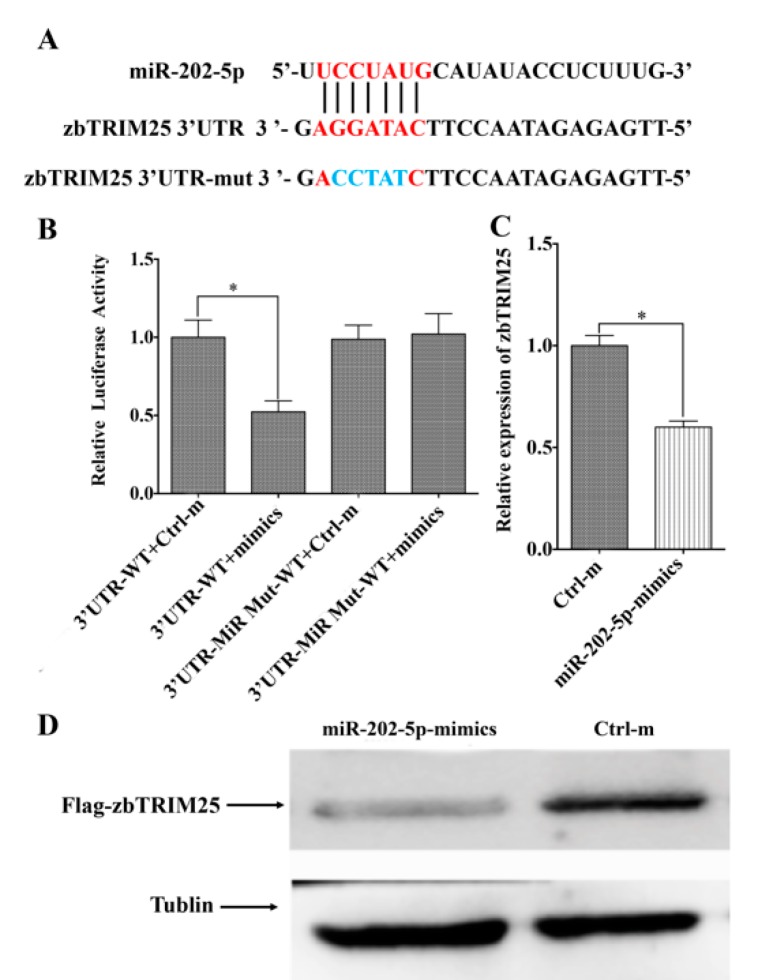
zbTRIM25 is the target of miR-202-5p. (**A**) The potential miR-202-5p binding sites in the zbTRIM25-3′UTR and its mutant. (**B**) miR-202-5p mimics and Ctrl-m were co-transfected into HEK 293T cells with luciferase reporter plasmid of wild-type zbTRIM25-3′UTR plasmid (3′UTR-WT) and mutant-type of zbTRIM25-3′UTR plasmid (3′UTR-miR Mut), respectively. (**C**) Overexpression of miR-202-5p in ZBE3 cells changes the *zbTRIM25* mRNA level as measured by qRT-PCR. Asterisks indicate the significant differences between groups (*: *p* < 0.05). (**D**), Enhanced miR-202-5p expression suppressed zbTRIM25 protein levels in HEK 293T cells. HEK 293T cells were co-transfected with miR-202-5p mimics or Ctrl-m and *pCMV-Flag-zbTRIM25* or *pCMV-Flag* vector for 48 h and harvested for a Western blot analysis, respectively.

**Figure 5:**
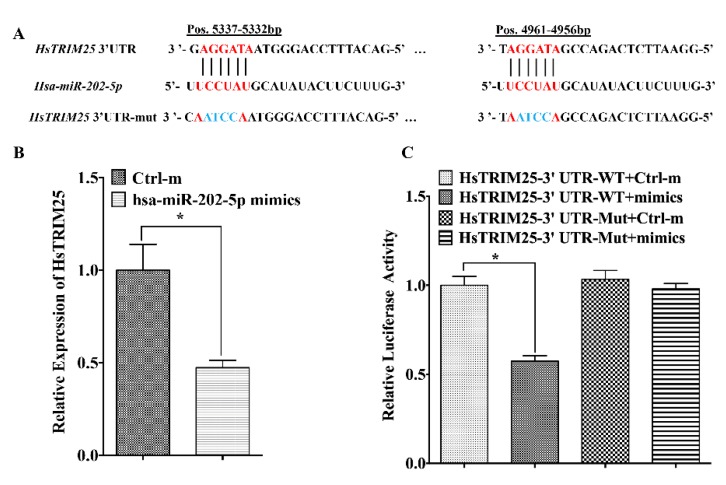
Human tripartite motif-containing protein (HsTRIM25) is targeted by hsa-miR-202-5p. (**A**) Two potential hsa-miR-202-5p binding sites in the HsTRIM25-3′UTR and its mutants. (**B**) Overexpression of hsa-miR-202-5p in HEK 293T cells changed the endogenous *HsTRIM25* mRNA level as measured by qRT-PCR. (**C**) The luciferase reporter vector encoding wild-type or mutated 3′ UTRs from HsTRIM25 was co-transfected into HEK 293T cells with hsa-miR-202-5p mimics or Ctrl-m, and luciferase activity was measured 48 h later. Asterisks indicate the significant differences between groups (*: *p* < 0.05).

To ascertain the mechanism through which miR-202-5p exerts the promotion role of RGNNV infection, the functional target genes of miR-202-5p were predicted with miRanda and Targetscan databases. Interestingly, zbTRIM25, which has been reported as a positive regulatory factor of RLRs signaling pathway [20], was predicted as a potential target of miR-202-5p (Figure 4A). Then, luciferase reporter assay was performed to confirm the possibility that zbTRIM25 was regulated post-transcriptionally by miR-202-5p. The relative activity of luciferase significantly decreased in zbTRIM25-3′UTR-WT and miR-202-5p mimics co-transfection group compared with that in the zbTRIM25-3′UTR-WT and Ctrl-m co-transfection group. However, no difference was found between the relative luciferase activities of zbTRIM25-3′UTR-mut and miR-202-5p mimics con-transfection group and zbTRIM25-3′UTR-WT and Ctrl-m co-transfection group (Figure 4B). To further validate zbTRIM25 as a target of miR-202-5p, its expression was examined in ZBE3 cells transfected with miR-202-5p mimics or Ctrl-m. As expected, the expression of zbTRIM25 was decreased significantly in miR-202-5p mimics transfected cells (Figure 4C). Moreover, in zbTRIM25 overexpressing HEK 293T cells, the zbTRIM25 protein level was also decreased by co-transfecting with miR-202-5p mimics (Figure 4D). Taken together, our results demonstrate that zbTRIM25 is an actual target of miR-202-5p. Interestingly, we found that the 3′UTR of human TRIM25 (HsTRIM25) contains two human miR-202-5p (hsa-miR-202-5p) binding sites (Figure 5A). Furthermore, endogenous *HsTRIM25* expression was significantly downregulated by hsa-miR-202-5p overexpression (Figure 5B), and the luciferase reporter assay demonstrated that hsa-miR-202-5p had a significant regulatory effect on the luciferase activity in hsa-miR-202-5p mimics and *HsTRIM25* co-transfected HEK 293T cells (Figure 5C). All these results demonstrated that hsa-miR-202-5p could target HsTRIM25.

### 3.4. MiR-202-5p Negatively Regulates zbTRIM25-Mediated RLRs Signaling Pathway

**Figure 6:**
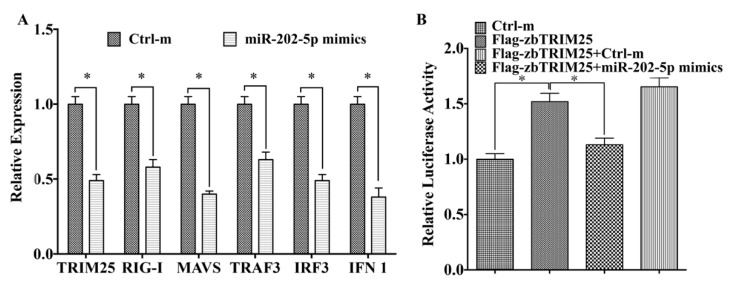
miR-202-5p suppresses zbTRIM25 expression and its downstream signaling. (**A**) Expression of zebrafish *TRIM25*, *RIG-I*, *MAVS*, *TRAF3*, *IRF3*, and *IFN 1* mRNA was detected by qRT-PCR in ZBE3 cells that were transfected with miR-202-5p mimics or Ctrl-m for 24 h. (**B**) HEK 293T cells were co-transfected with *pGL3-DrIFN 1-pro-Luc*, *pCMV-Flag-zbTRIM25,* and miR-202-5p mimics or Ctrl-m for 48 h, respectively, and luciferase activity was measured. Asterisks indicate the significant differences between groups (*: *p* < 0.05).

Given that miR-202-5p targets zbTRIM25 and negatively regulates its expression, we examined whether miR-202-5p could suppress zbTRIM25-mediated RLRs signaling pathway. As shown in Figure 5, the expression levels of *zbTRIM25* and its downstream genes, *RIG-I*, *MAVS*, *TRAF3*, *IRF3*, and *IFN 1* decreased to some different extent at 24 h post RGNNV infection in miR-202-5p mimics transfected ZBE3 cells (Figure 6A). In addition, reporter gene analyses showed that miR-202-5p mimics significantly inhibited zbTRIM25 mediated IFN activities (Figure 6B). These findings suggested that miR-202-5p suppressed zbTRIM25-mediated RLRs signaling pathway.

### 3.5. MiR-202-5p Suppresses zbTRIM25-Mediated zbRIG-I Activation

**Figure 7:**
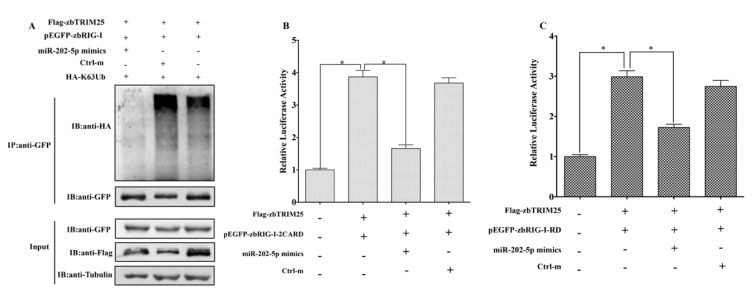
miR-202-5p inhibits zbTRIM25-mediated zbRIG-I ubiquitination and interferon (IFN) promoter activity. (**A**) HEK 293T cells were transfected with plasmids and miR-202-5p mimics or Ctrl-m as indicated for 24 h. At 24 h after poly I:C treatment, cells were lysed, and the cell lysates were either analyzed directly by using anti-GFP, anti-Flag, and anti-Tubulin antibodies via Western blotting (input) or subjected to immunoprecipitation using anti-GFP antibodies. The precipitates (IP) were analyzed by Western blotting with anti-GFP and anti-HA antibodies, respectively. HEK 293T cells were transfected with *pCMV-Flag* or *pCMV-Flag-zbTRIM25*, miR-202-5p mimics or Ctrl-m together with *pEGFP-zbRIG-I-2CARD* (**B**) or *pEGFP-zbRIG-I-RD* (**C**) as well as *pGL3-DrIFN 1-pro-Luc* and *pRL-TK* plasmids. Luciferase activities were measured and normalized to the amount of Renilla luciferase activities. Data represent the mean ± SD (*n* = 3). Asterisks indicate the significant differences between groups (*: *p < 0.05*).

We have confirmed that zbTRIM25 promoted K63 ubiquitination of zbRIG-I and enhanced zbRIG-I’s IFN-inducing activities [20]. Given zbTRIM25 was a miR-202-5p target gene, we hypothesized that miR-202-5p might suppress zbTRIM25 mediated zbRIG-I activities to negatively regulate RLRs signaling pathway. To test this hypothesis, we first examined the effect of miR-202-5p on zbTRIM25-mediated zbRIG-I ubiquitination. As expected, the level of ubiquitination of zbRIG-I was inhibited by miR-202-5p mimics transfection (Figure 7A). Subsequently, reporter gene analyses showed that zbTRIM25 mediated zbRIG-I-2CARD and zbRIG-I-RD’s IFN-inducing activities were significantly reduced by miR-202-5p mimics (Figure 7B,C). Therefore, miR-202-5p inhibited RLRs signaling through targeting zbTRIM25.

## 4. Discussion

The RLRs signaling pathway plays pivotal roles in virus recognition and initiation of the antiviral immune response. To survive in host cells, various viruses have developed different strategies for their evading and subverting of the immune responses. Previous reports have demonstrated that NNV can evade host innate immunity and causes persistent infection in many fish species, including zebrafish [11]. However, to date, knowledge about the immune evasion strategies of NNV is very limited. Increasing evidence has shown that viruses have evolved a wide variety of mechanisms to evade the host immune systems through interfering with the RLR signaling [3,24]. Several studies have exhibited the relationship between viral infection-induced miRNAs and RLRs signaling pathway, and demonstrated many miRNAs functioned as negative regulators of RLRs signaling pathway during viral infection. For example, miR-3570 inhibited the RIG-I-dependent innate immune response to rhabdovirus by targeting MAVS in miiuy croaker [16]. MiR-22 negatively regulated type I IFN production by targeting MAVS [25]. A previous study has shown that miRNAs were associated with RGNNV infection [26], but the exact functions and regulation mechanisms of miRNAs during RGNNV infection are poorly understood. Herein, miR-202-5p expression was up-regulated in vivo and in vitro during RGNNV infection, suggesting its close association with RGNNV infection. Many studies have demonstrated that miR-202-5p is an evolutionarily conserved miRNA associating with gonad development in vertebrates [22,27,28]. Recently, we reported that miR-202-5p regulated primordial germ cell migration by directly targeting Cdc42se1 [17]. In this study, we investigated a novel role of miR-202-5p in the immune response to RGNNV infection. Firstly, we examined the effect of miR-202-5p on host antiviral signaling. Our results showed that miR-202-5p promoted IFN and ISGs inhibition caused by RGNNV infection in vitro. Furthermore, in vitro and in vivo experiments corroborated that downregulation of miR-202-5p could inhibit RGNNV replication. All these data confirmed miR-202-5p was a positive mediator of immunosuppression and had an enhanced effect on RGNNV replication in zebrafish. To the best of our knowledge, this is the first study to describe the role of miR-202-5p during virus infection.

To gain an insight into the precise mechanisms by which miR-202-5p regulated immune response during RGNNV infection, its target genes were predicted by using the bioinformatics tool. zbTRIM25 was predicted as one of its target genes, and subsequently was confirmed by a series of experiments. Moreover, we further confirmed that hsa-miR-202-5p also targeted human TRIM25, demonstrating the universality of our findings. In this study, we found that miR-202-5p could suppress zbTRIM25-mediated RLRs signaling pathway, indicating that miR-202-5p might negatively regulate zbTRIM25 expression, thereby promoted RGNNV infection. 

Increasing evidence indicates a crucial role of post-translational modifications in modulating TRIM25/RIG-I signaling. TRIM25 has been shown to positively regulate innate antiviral response by inducing the K63-linked ubiquitination of RIG-I [29]. Similar to other TRIM proteins, both gene expression and protein abundance of TRIM25 are tightly regulated and some factors that regulated its expression have been reported. For instance, the C-terminal zinc-fingers of ZCCHC3 interacted with the C-terminal SPRY domain of TRIM25 and was important for K63-linked polyubiquitination and activation of RIG-I and MDA5 mediated by TRIM25 [30]. Ubiquitin-specific protease 15 interacted with and deubiquitylated TRIM25, thereby promoting RIG-I-mediated antiviral signaling during viral infection [31]. C-Src induced TRIM25 tyrosine phosphorylation which facilitated TRIM25-mediated RIG-I ubiquitination [32]. Influenza A virus nonstructural protein 1 (NS1) specifically inhibits TRIM25-mediated RIG-I-CARD ubiquitination through its interaction with the coiled-coil domain of TRIM25, thereby suppressing RIG-I signal transduction [33]. Here, miR-202-5p was identified as a negative regulator of zbTRIM25, which will contribute to the understanding of the regulation mechanism underlying TRIM25-dependent innate immunity.

**Figure 8:**
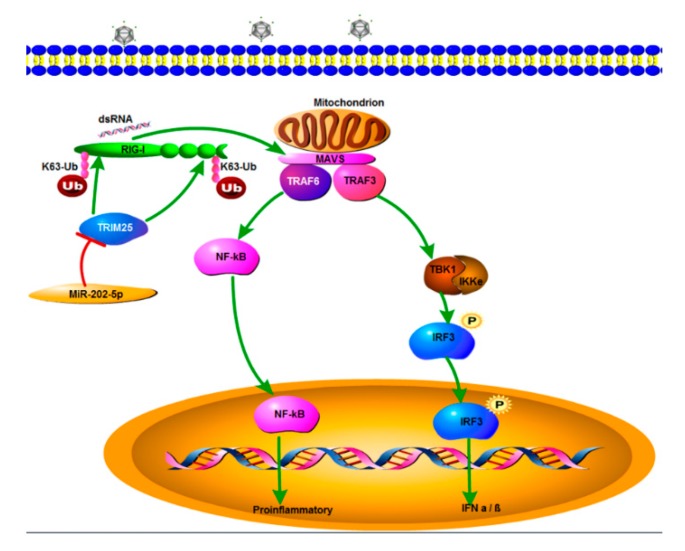
A schematic model of miR-202-5p-mediated RIG-I-like receptors (RLRs) signaling pathway during viral infection. miR-202-5p targets zbTRIM25 and inhibits zbTRIM25-mediated zbRIG-I ubiquitination, thereby negatively regulating RLRs signaling pathway during RGNNV infection.

MiRNAs are well known for their post-transcriptional regulation of gene expression, and accumulated information has revealed that several miRNAs are involved in immune regulation via targeting the key components of intracellular pathogen sensing pathways [34]. In our current study, zbTRIM25 was proven to be a target gene of miR-202-5p and miR-202-5p inhibited zbTRIM25-mediated IFN 1 promoter activation and K63-linked ubiquitination of zbRIG-I. Thus, we speculated that miR-202-5p attenuated the K63-linked ubiquitination of zbRIG-I by targeting zbTRIM25, which subsequently might hinder zbRIG-I and MAVS interaction, and thereby inhibited the antiviral signaling (Figure 8). It has been reported that viral infections can modulate the expression of several miRNAs, which in turn regulate RLRs-mediated IFN activation. For example, Enterovirus 71 3C protein inhibited the expression of miR-526a, leading to the upregulation of cylindromatosis, which negatively regulates RLRs-mediated IFN production [35]. Here, we reported that RGNNV infection induced the expression of miR-202-5p, however, the mechanism underlying miR-202-5p transcription activation by RGNNV remains unknown. Whether and how RGNNV proteins participate in miR-202-5p induction needs to be further studied.

In summary, miR-202-5p was identified as a mediator of RLRs signaling pathway and promoted RGNNV infection by directly targeting zbTRIM25, with subsequent inhibition of zbTRIM25-mediated zbRIG-I ubiquitination and activation of IFN production (Figure 8). These findings represent a new mechanism underlying RGNNV evasion of the host innate immune system.

## Supplementary Materials

The following are available online at https://www.mdpi.com/1999-4915/12/3/261/s1, Table S1: Primers used in this study.
