# Supplementary material for: MiR-202-5p Inhibits RIG-I-Dependent Innate Immune Responses to RGNNV Infection by Targeting TRIM25 to Mediate RIG-I Ubiquitination
Source: Viruses. 2020 Feb 27;12(3):261. doi: 10.3390/v12030261 (PMC7150862; doi:10.3390/v12030261)
Supplement: Supplementary file 1 [file viruses-12-00261-s001.pdf]

Table S1. Primers used in this study

Primer name	Sequences (5'-3')	Comment
18s rRNA-F	TCGCTAGTTGGCATCGTTTATG	QPCR of 18s rRNA
18s rRNA-R	CGGAGGTTCGAAGACGATCA	QPCR of 18s rRNA
NNV-RDRP-F	GCTTTATGCGTGAGTGCGTC	QPCR of RDRP
NNV-RDRP-R	GCTGTTTCCGTCTGTTGTGAG	QPCR of RDRP
MiR-202-5p-RT	TTCCTATGCATATACCTCTTTG	QPCR of miR-202-5p
MRQ-3'-R	TTTTTTTTTTTTTTTTTT	QPCR of miR-202-5p
zbTRIM25-3'UTR-MUT-F	CCTTCGCGAGAGTGACAG	psiCHECK2-zbTRIM25-3'UTR-MUT
zbTRIM25-3'UTR-MUT-R	GTCACTCTCGCGAAGGTT	psiCHECK2-zbTRIM25-3'UTR-MUT
zbTRIM25-3'UTR-F	ATAAGAATGCGGCCGCCACTGTGGCAACCCCAGAT	psiCHECK2-zbTRIM25-3'UTR
zbTRIM25-3'UTR-R	CCGCTCGAGTTATTTGGTTGGTCATTT	psiCHECK2-zbTRIM25-3'UTR
zbTRIM25-F	CCGCTCGAGGTGCGGAACAAATGTCTCTG	pCMV-Flag-zbTRIM25
zbTRIM25-R	ATAAGAATGCGGCCGCCAAACAGAGCAAGTCTTT	pCMV-Flag-zbTRIM25
HsTRIM25-3'UTR-F	CCGCTCGAGCAGACCGATAGGATGTC	psiCHECK2-zbTRIM25-3'UTR
HsTRIM25-3'UTR-R	ATAAGAATGCGGCCGCACACTCCTATTACCCTGG	psiCHECK2-zbTRIM25-3'UTR
HsTRIM25-3'UTR-MUT-F	CCGCTCGAGCAGACCGTATCCTTGTC	psiCHECK2-HsTRIM25-3'UTR-MUT
HsTRIM25-3'UTR-MUT-R	ATAAGAATGCGGCCGCACACAGGATATACCCTGG	psiCHECK2-HsTRIM25-3'UTR-MUT
zbTRIM25-F	GACGACCCTGCGAACTATCC	QPCR of zbTRIM25
zbTRIM25-R	CCAGCAAGATGCCAACACG	QPCR of zbTRIM25
zbISG15-F	AAAACTGCTTGGCGGTGA	QPCR of ISG15
zbISG15-R	GGAGTTCAGCCCGTAGCTGC	QPCR of ISG15
zbPKR-F	AGGTTTACTATGAGAGCTAT	QPCR of PKR
zbPKR-R	ACTCGCAGGCCTTGAACA	QPCR of PKR
zbIFN-F	GCTCTGCGTCTACTTGCGAA	QPCR of IFN 1
zbIFN-R	GCGGCTTGGAAATGGTGT	QPCR of IFN 1
zbMXa-F	ATGACCCAGCGGAAGTGG	QPCR of MXa
zbMXa-R	GTCAACATTGCATGGCACCA	QPCR of MXa
zbIRF3-F	CAAAACCGCTGTTCGTGCC	QPCR of IRF3
zbIRF3-R	TCGTCGCTGTTGGAGTCCTG	QPCR of IRF3
zbMAVS-F	CAGGCGAGAGAACTGGATTG	QPCR of MAVS
zbMAVS-R	CAGGTGGCATTAGCAGAGGAA	QPCR of MAVS
zbRIG-I-F	CGCCAGTCCTGATAAACCCA	QPCR of RIG-1
zbRIG-I-R	GCAGCGGCAGATCCTGTATG	QPCR of RIG-I
zbTRAF3-F	ATGTCCGCAGGGCGTAAT	QPCR of TRAF3
zbTRAF3-R	ACACTCAGTCTGGCGTGGGT	QPCR of TRAF3
